# Copy number variation of scavenger-receptor cysteine-rich domains within *DMBT1* and Crohn's disease

**DOI:** 10.1038/ejhg.2015.280

**Published:** 2016-01-27

**Authors:** Shamik Polley, Natalie Prescott, Elaine Nimmo, Colin Veal, Ida Vind, Pia Munkholm, Peder Fode, John Mansfield, Paal Skyt Andersen, Jack Satsangi, Christopher G Mathew, Edward J Hollox

**Affiliations:** 1 Department of Genetics, University of Leicester, Leicester, UK; 2 Department of Medical and Molecular Genetics, King's College London, London, UK; 3 Centre for Genomic and Experimental Medicine, University of Edinburgh, Edinburgh, UK; 4 Microbiology and Infection Control Unit, Statens Serum Institut, Copenhagen, Denmark; 5 Institute of Genetic Medicine, Newcastle University, Newcastle upon Tyne, UK; 6 Sydney Brenner Institute for Molecular Bioscience, University of the Witwatersrand, Johannesburg, South Africa

## Abstract

Previous work has shown that the gene *DMBT1*, which encodes a large secreted epithelial glycoprotein known as salivary agglutinin, gp340, hensin or muclin, is an innate immune defence protein that binds bacteria. A deletion variant of *DMBT1* has been previously associated with Crohn's disease, and a *DMBT1*^−/−^ knockout mouse has increased levels of colitis induced by dextran sulphate. *DMBT1* has a complex copy number variable structure, with two, independent, rapidly mutating copy number variable regions, called CNV1 and CNV2. Because the copy number variable regions are predicted to affect the number of bacteria-binding domains, different alleles may alter host–microbe interactions in the gut. Our aim was to investigate the role of this complex variation in susceptibility to Crohn's disease by assessing the previously reported association. We analysed the association of both copy number variable regions with presence of Crohn's disease, and its severity, on three case–control cohorts. We also reanalysed array comparative genomic hybridisation data (aCGH) from a large case–control cohort study for both copy number variable regions. We found no association with a linear increase in copy number, nor when the CNV1 is regarded as presence or absence of a deletion allele. Taken together, we show that the *DMBT1* CNV does not affect susceptibility to Crohn's disease, at least in Northern Europeans.

## Introduction

Crohn's disease (CD) is a chronic debilitating inflammatory disease that most frequently affects the terminal ileum but can affect any part of the gastrointestinal tract. In the West, CD has a prevalence of ~150 per 100 000,^1^ with environmental and genetic variations making an approximately equal contribution to disease risk.^2^

The most recent progress in elucidating the genetic variation responsible for CD has come from SNP-based genome-wide association studies, which have identified 163 loci that contribute to the genetic risk of the disease.^3, 4^ Nevertheless, even with well-powered analyses of 15 000 cases and 15 000 controls, only 13.6% of disease variance has been explained, suggesting that other genetic risk variants exist that are not interrogated by current SNP-GWAS approaches. Copy number variation, where a whole or part of a gene differs in copy number in different individuals, is a potential candidate type of variation that is often not well-tagged by flanking SNP alleles.^5^ CNV of the beta-defensin genes and amylase has been shown to affect susceptibility to psoriasis and obesity, respectively,^6, 7^ indicating that CNV can contribute to genetic variance of common complex diseases. A genome-wide association study directly interrogating CNV by arrayCGH identified a CNV in the HLA region and at the *IRGM* gene associated with CD,^8^ but other more complex CNVs may also be associated with CD.

Because methods to reliably and cost-effectively type CNV genome-wide are lacking, recent literature has focused primarily on studies of candidate genes chosen for their known role in the aetiology of CD. One example is association of CNV of the beta-defensin gene region with CD, where an initial study supported an association of low beta-defensin copy number with CD, an effect only seen in colonic CD rather than ileal CD.^9^ A subsequent study on a larger cohort of cases and controls found a significant association but in the reverse direction,^10^ and both studies are limited by low statistical power and limitations in the technology used to type CNV.^11, 12^ Indeed, the large genome-wide arrayCGH association study of CD patients and controls found no evidence of association with beta-defensin CNV,^8^ a finding supported by a rigorous study, which also showed that real-time quantitative PCR methods often used to type CNV could easily generate false-positive associations of CNV and disease.^11^ This emphasised the importance of robust copy number detection methods that minimised the chance of false-positive results.

Another CNV that has been associated with CD is a deletion of part of the *DMBT1* gene, called *DMBT1*^*SR47*−^.^13^ CD patients have been shown to have a higher frequency of this deletion compared to controls. *DMBT1* is a particularly attractive candidate gene, as it encodes a large secreted glycoprotein (also known as salivary agglutinin, gp340, hensin or muclin) that is expressed in the gastrointestinal tract and is upregulated in CD.^14^ Furthermore, DMBT1 binds a wide variety of Gram positive and Gram negative bacteria, at least in saliva and the lung,^15, 16^ and *DMBT1* knockout mice show subtly enhanced sensitivity to experimentally induced colitis,^13^ although this has not been confirmed with an alternative *DMBT1* knockout mouse.^17^ Nevertheless, the evidence suggests that *DMBT1* has an important innate defence function in the gastrointestinal tract.

**Figure 1:**
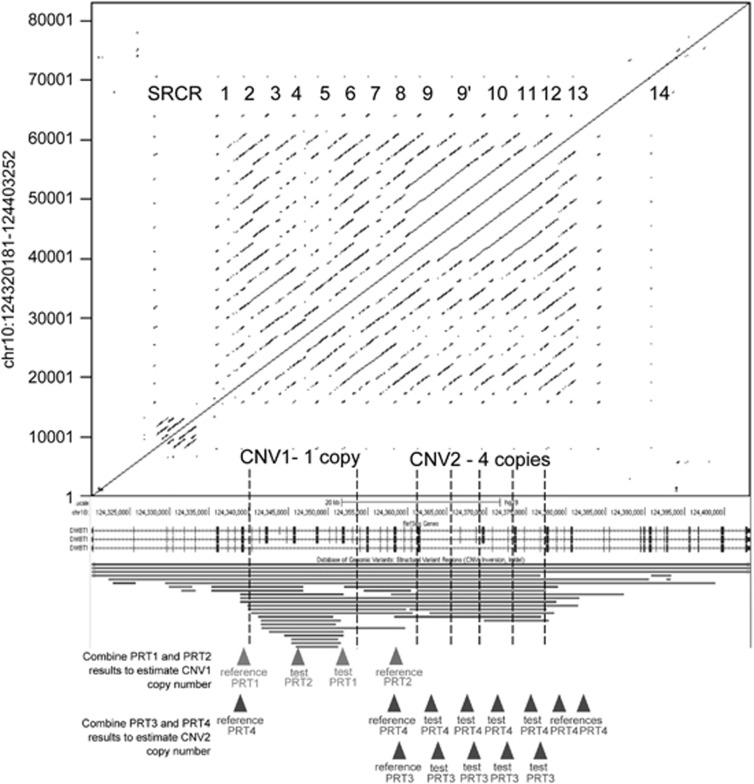
Overview of the copy number variation at *DMBT1*. A dotplot shows the repeated nature of the *DMBT1* gene (shown from a screenshot from the UCSC genome browser). The tandemly arranged SRCR repeat regions are shown, including SRCR14, which does not bind bacteria. The genome assembly shows one assembled copy of CNV1 and four assembled copies of CNV2. CNV regions, as recorded in the Database of Genome Variants, are shown below the *DMBT1* gene structure. Below these, location of reference and test amplicons of the four independent paralogue ratio tests (PRTs) that measure copy number of CNV1 and CNV2 are shown.

The canonical DMBT1 protein is composed of a regular array of 13 scavenger-receptor cysteine-rich (SRCR) domains interspersed with SID domains, and followed by a CUB domain, a diverged SRCR domain, a further CUB domain and then finally a zona pellucida domain.^18^ The polymorphic *DMBT1*^*SR47*−^ deletion previously associated with CD leads to the loss of four SRCR domains (SRCR3-6),^19, 20^ Figure 1, and since these SRCR domains have been shown to contain the binding sites for bacteria,^15^ it has been suggested that the deletion leads to a quantitative change in the ability of DMBT1 to bind bacteria, limiting the protection of the host mucosa against intestinal flora and therefore contributing to the pathogenesis of CD.^13^

Recent work has demonstrated that the *DMBT1*^*SR47*−^ polymorphic deletion is in fact part of a wide spectrum of alleles affecting the copy number of SRCR domains within *DMBT1*^20^ (Figure 1). Specifically, at the locus where the polymorphic deletion occurs (termed CNV1), there is also a polymorphic duplication allele of the same 4-SRCR domain repeat unit. Furthermore, at the C-terminal SRCR region there is a further CNV (termed CNV2) where a single SRCR domain unit can vary between 0 and 11 copies per diploid genome. Taken together, this indicates that although the canonical *DMBT1* 13-SRCR array structure represents a common genotype containing 26 tandemly arrayed SRCR domains per diploid genome (2 × 13-SRCR arrays), in reality a wide range of SRCR domain numbers have been observed within *DMBT1* ranging from 14 to 40 SRCR domains per diploid genome. Therefore, through allelic variation alone, DMBT1 molecules have the potential to contain between 7 and 20 SRCR domains, as a conservative estimate.^20^

Given this extensive variation, and the robust methods used to type it on small amounts of genomic DNA, we endeavoured to first replicate the original observation of an association of SRCR copy number on CD on three large Northern European case–control cohorts, and to extend the analysis to the full allelic spectrum of *DMBT1* SRCR domain variation, which might explain a significant amount of the genetic variance in CD susceptibility.

## Methods

### Danish cohort

DNA was extracted from the peripheral blood of native Danish CD patients recruited from a well-defined geographical region (Copenhagen capital area, Denmark) during a 2-year period from 1 January 2003 to 31 December 2004. The details of the Danish CD cohort are described elsewhere.^21, 22^ DNAs from healthy blood donors from the Danish national blood bank were included as controls.

### Scottish cohort

DNA was isolated from the peripheral blood from CD patients were collected at the Western General Hospital, Edinburgh, Scotland, which is a tertiary referral centre for inflammatory bowel disease (IBD) in South-East Scotland. Detailed description of the Scottish cohort is given elsewhere.^11^ Written consent from CD patients was obtained prior to inclusion in the study. DNA from blood samples from unrelated spouses/friends of IBD patients or samples obtained from the Scottish Blood Transfusion Service were used as healthy controls. The study protocol was approved by the Medicine and Oncology Subcommittee of the Lothian Local Research Ethics Committee (LREC 2000/4/192).

### English cohort

White European patients with CD were recruited from specialist IBD clinics in London and Newcastle as reported elsewhere^23^ after informed consent and ethical review (REC 05/Q0502/127). Patients were recruited from Guy's and St Thomas' Hospitals London, United Kingdom, St Mark's Hospital London, United Kingdom, and the Royal Victoria Infirmary, Newcastle, United Kingdom after ethical review and informed consent from CD patients. Human random control (HRC) DNA samples from lymphoblastoid cell lines derived from UK individuals (from the ECACC collection held by Public Health England: http://www.phe-culturecollections.org.uk/) were used as control samples.

### Copy number typing

We used our extensively validated and robust paralogue ratio test approach to type copy number on genomic DNA samples, as described previously.^20, 24^ Briefly, test and reference amplicons are generated using the same PCR primer pair, one primer fluorescently labelled. The primers are designed so that test and reference amplicons can be distinguished by a small difference in product length by capillary electrophoresis on an ABI 3100xl (Applied Biosystems/Life Technologies, Paisley, UK). Eight positive control DNAs from the HapMap panels were run on every plate to act as calibrators (Supplementary Table 1). Our previous study, using repeat testing of identical samples, estimates the experimental error rate of CNV1 determination to be 0.37% and of CNV2 to be 0.33%.^20^ WTCCC data was provided courtesy of the WTCCC Access Committee and Dr Matthew Hurles (Wellcome Trust Sanger Institute). All data have been deposited with dbVar http://www.ncbi.nlm.nih.gov/dbvar accession number nstd77.

### Statistical analysis

Raw copy number data from PRT was normalised to have a SD of one across the cohort. Data from cases and controls were analysed together, and, following visual inspection of a histogram of the raw data, a Gaussian mixture model (GMM) fitted to the data with the number of components (individual Gaussian distributions corresponding to each copy number) of the model determined by inspecting the number of peaks in the histogram, and by previous knowledge of the range of copy number variation in cohorts described previously. The variance of each Gaussian distribution was assumed to be the same when fitting the models to the data. Fitting the GMM allows integer copy numbers to be called from the data with an associated Bayesian posterior probability value for each call. It also allows fitting of two different models for cases and controls to provide a formal test of association of copy number with disease.^25^ This analysis is implemented in the R package CNVtools v 1.42.3 (www.bioconductor.org). Examples of GMM fits to the data are shown in Supplementary Figure 1, for PRT data.

The raw aCGH data for the CD cohort was normalised two different ways: named as normalised1 and normalised2. In case of first normalisation (normalised1), the log of the ratio of the red and green channel data (log(R/G)) was used whereas in second normalisation (normalised2), the log of the ratio of the quantile-normalised red and green channel data (log(QNorm(R)/QNorm(G))) was calculated. Data from 12 probes spanning CNV1 and 18 probes spanning CNV2 were summarised using the first principal component of the data, so that each sample had one summary value for CNV1 and one summary value for CNV2. For CNV1, plotting the data on a histogram gave three clusters, and CNV1 copy number was called using a three-component GMM. For some samples, duplicate aCGH data were available. In such cases, the duplicate sample with the lowest posterior probability in support of an integer copy number was removed prior to case–control analysis. Normalised1 data were used for CNV1 case–control analysis, and normalised2 data were used for CNV2 analysis. Examples of GMM fits to the data are shown in Supplementary Figure 2, for aCGH data.

Fisher's exact tests and regression analyses were performed using R 3.1.0 (https://www.r-project.org/), and meta-analysis used the R package meta v4.3. All cohorts had a power of >0.9 to detect the effect size previously observed^13^ at a significance level of 0.05 or below, with the exception of the Danish cohort, which had a power of 0.76.

## Results

We genotyped 1449 cases and 994 controls from the English, Scottish and Danish cohorts using our paralogue ratio test approach, described previously. The copy number distribution of CNV1 ranged from 0 to 4 in all populations, and CNV2 ranged from 1 to 11 (Supplementary Figures 1 and 2), which is consistent with previous studies on European populations.^20^ Histograms of the raw data show clear clustering for CNV1 but poorer clustering for CNV2, where clear histogram peaks are seen only for lower copy numbers. Furthermore, visual inspection shows that for CNV2 calling quality varied from cohort to cohort, and this is reflected in the quality score of the GMM (Q; Supplementary Table 2) that is fitted to the data and used to call integer copy numbers. Because of this, we used two approaches for testing for association with disease. First, we called integer copy number using CNVtools and used those copy numbers in a standard Fisher's exact test. Second, we used a feature of CNVtools that tests for association at the same time as fitting the GMM, which has the advantage of explicitly taking into account uncertainty in copy number calling.

**Figure 2:**
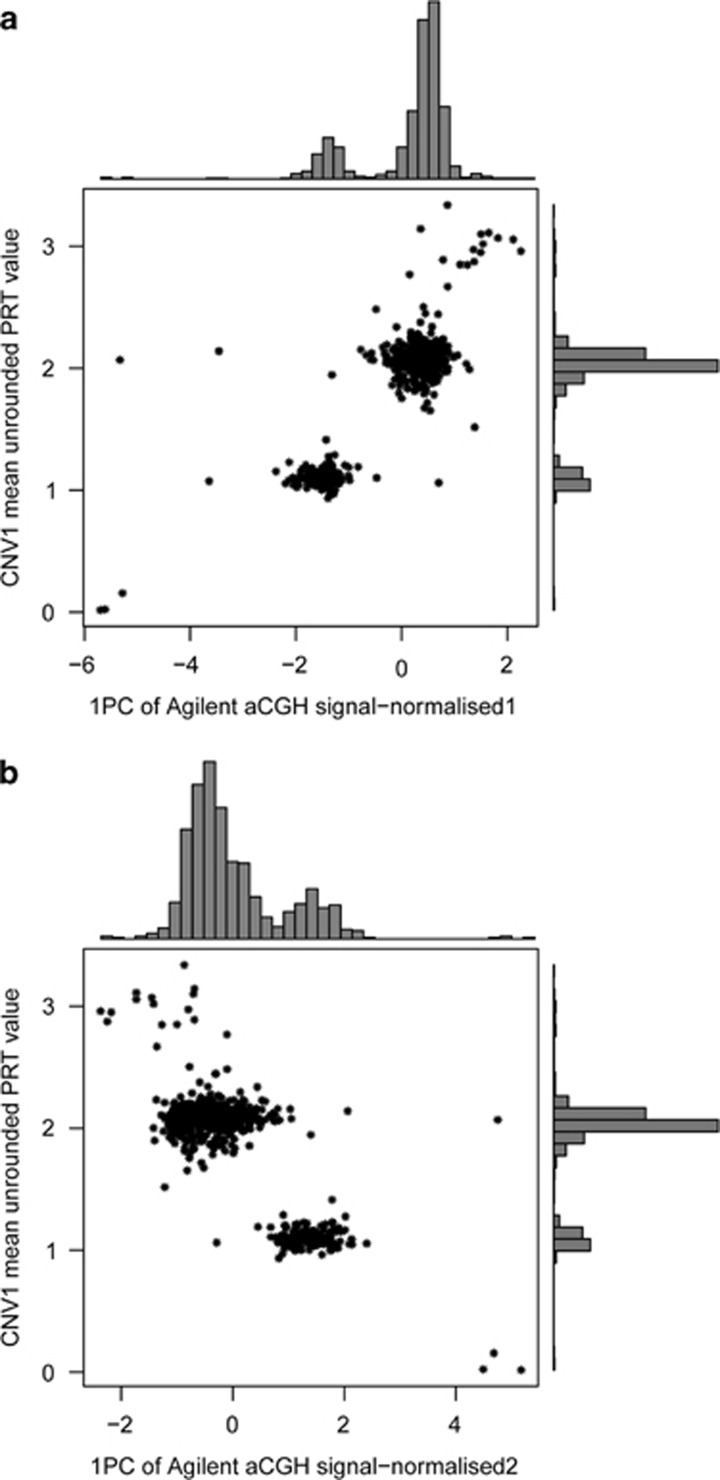
Analysis of calling CNV1 copy number using PRT and arrayCGH. (**a**) Six hundred and eighty eight samples from the English CD cohort and 97 samples from the Scottish CD cohort with copy number measured by both PRT (*y* axis) and aCGH (*x* axis). aCGH data here are normalised using log(R/G) and represent the first principal component value of 12 probes. (**b**) As above but with aCGH, data here are normalised using log(QNorm(R)/QNorm(G)), where Qnorm is quantile-normalised.

**Figure 3:**
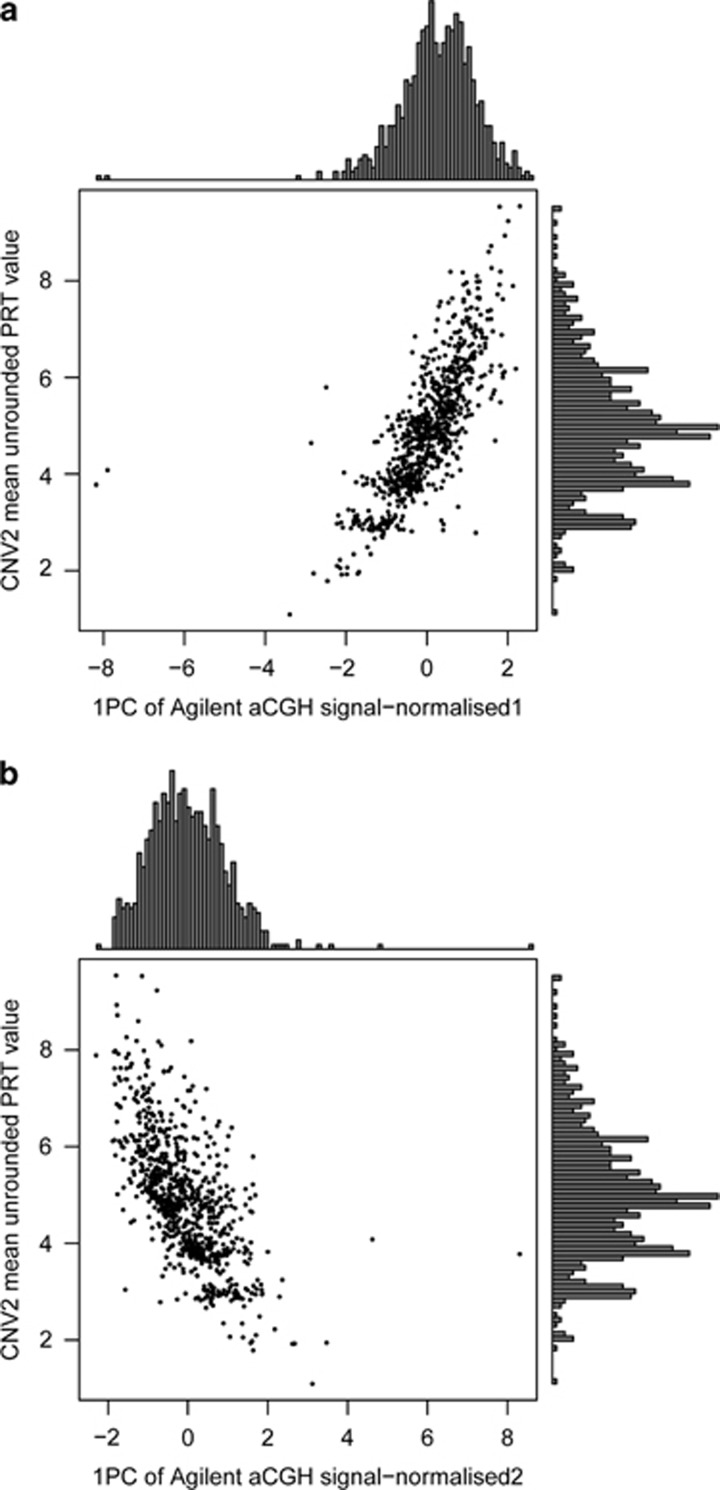
Analysis of calling CNV2 copy number using PRT and arrayCGH. (**a**) Six hundred and eighty eight samples from the English CD cohort and 97 samples from the Scottish CD cohort with copy number measured by both PRT (*y* axis) and aCGH (*x* axis). aCGH data here are normalised using log(R/G) and represent the first principal component value of 18 probes. (**b**) As above but with aCGH, data here are normalised using log(QNorm(R)/QNorm(G)), where Qnorm is quantile-normalised.

A subset of both the English and Scottish cohorts had been analysed by arrayCGH as part of the Wellcome Trust Case Control Consortium genome-wide CNV analysis. This allowed us to compare our copy number calling using PRT with DNA dosage data generated by arrayCGH. The arrayCGH data and PRT raw data were correlated for both CNV1 (*r*^2^=0.75 using normalised1 data; *r*^2^=0.65 using normalised2 data) and for CNV2 (*r*^2^=0.55 using normalised1 data; *r*^2^=0.43 using nomalised2 data). Figure 2 shows that summarising the aCGH data as the first principal component of 12 probes spanning CNV1 gives concordance with PRT results, and suggests that these aCGH data could call CNV1 copy number quite robustly. Compared to the normalised2 approach (Figure 2b; see Methods), data normalised using the normalised1 approach showed a stronger correlation with the PRT raw data and showed better distinction between the two main peaks (copy numbers 1 and 2; Figure 2a). Therefore, the normalised1 data were chosen for the full cohort analysis. For CNV2, although aCGH measures the DNA dosage and is correlated with PRT calls, there is a single continuous distribution with no evidence of clustering about integer copy numbers (Figure 3), and the correlation with PRT raw data is much weaker than in the case with the CNV1 data.

**Figure 4:**
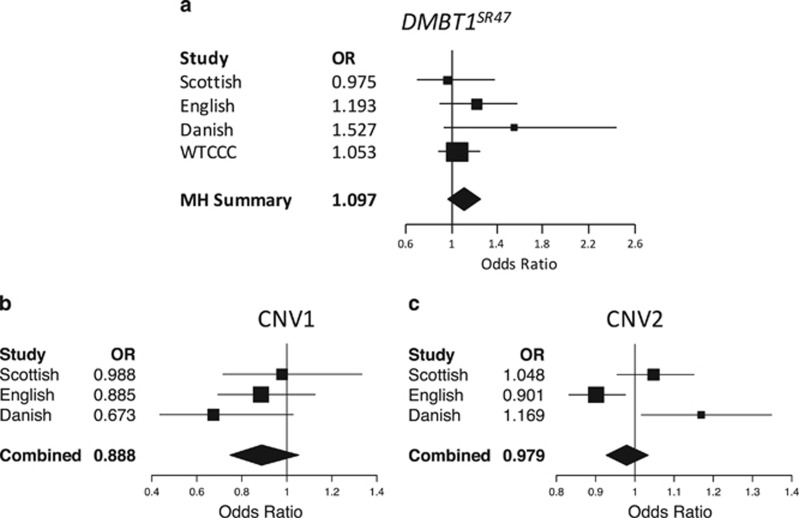
Meta-analysis of cohorts in the association study. Forest plots of odds ratios for the deletion variant of *DMBT1*^SR47^ for Scottish, English, Danish and WTCCC data sets only (**a**), Forest plots of the odds ratio per copy for CNV1 (**b**) and CNV2 (**c**). Each diagram displays the odds ratios for each data set as a box with the 95% confidence interval marked by lines. The ‘MH Summary' represents the 95% confidence interval of the Mantel-Haenszel combined odds ratio for all data sets, whereas ‘Combined' represents the 95% confidence interval for totals for CNV1 and CNV2.

For the first test of association of copy number with CD, we followed the approach described in Renner *et al.* In that paper CNV1 was genotyped as the deletion *DMBT1*^*SR47*−^ using a PCR-based approach. Previously, we have shown that this is a simplification of the CNV, with duplications also being observed in the population.^20^ Copy number 0 is equivalent to a homozygous deletion *DMBT1*^*SR47*−/−^, 1 to a heterozygous deletion *DMBT1*^*SR47*+/−^and 2 to a homozygous reference *DMBT1*^*SR47*+/+^. Copy numbers 3 and 4 represent heterozygous and homozygous duplications, respectively. To directly compare our data with the previously published data, we called deletion genotype from our CNV1 data, grouping all CNV1 copy numbers of 2 and above as homozygous reference genotype. We also called deletion genotype from the WTCCC aCGH data from samples not included in our English and Scottish cohorts. For the 785 samples where we had matching PRT and arrayCGH data, six samples disagreed for the genotype called, giving a discordance rate of <1.6% (upper 95% confidence limit). All 785 samples with matching PRT data were removed from the WTCCC cohort analysis. Analysis of allele frequency counts in each cohort showed a higher frequency of the deletion allele in CD patients in three of the four cohorts, but the differences were not statistically significant (Mantel-Haenszel OR 1.10, 95% CI 0.97–1.24, *P*=0.40; Table 1; Figure 4a).

We then asked whether full copy number typing of CNV1, where higher copy numbers corresponding to duplications can be called, would strengthen our association. Unfortunately, aCGH did not call high copy numbers effectively (Figure 2; Supplementary Figure 2), and so we were limited to the cohorts typed by PRT. By using logistic regression to test the linear effect on CD case–control status with each increase in copy number, we found no significant effect (combined *P*=0.17; Table 2; Figure 4b), a result confirmed when analysed using the likelihood approach of CNVtools (Supplementary Table 2). This suggests that the duplication allele at CNV1 is unlikely to protect against CD, although given the low frequency of this allele we may not have power to detect anything but a strong effect.

We then examined whether copy number at CNV2 was associated with CD. Analysis of our three cohorts provided apparently contradictory results, with the Scottish cohort showing no association, the English cohort showing a marginally higher copy number (*P*=0.01) in the CD patients and the Danish cohort showing a marginally lower (*P*=0.03) copy number in the CD patients (Table 3; Supplementary Table 3). This variation is due to variation in the patients rather than the controls, as the mean copy number in the controls is remarkably consistent across all three cohorts (Table 3). The simplest interpretation of the results is that of stochastic variation about a null result, and indeed combining the data sets suggest the following: combined *P*=0.446, Mantel-Haenszel OR=0.98, 95% CI 0.927–1.034 (Figure 4c). It may be the case that batch effects in typing high copy numbers of this CNV have generated this inconsistency. Indeed, even carefully designed CNV studies are prone to batch effects and the Scottish cohort was the only cohort where cases and controls originated from the same laboratory, and were randomly distributed across all experimental plates.

For the Scottish cohort, age of CD first diagnosis data were available as a proxy for age of onset, and it is conceivable that CNV of the SRCR domains within *DMBT1* could affect this trait, notwithstanding an overall effect on risk of developing CD. We analysed the effect of copy number at both CNV1 and CNV2 with age at diagnosis (Table 4), controlling for the known effect of sex on age of onset. We confirmed that females have on average a later age of onset in this cohort, but found no evidence of an effect of CNV1 or CNV2. Analysis of CNV1 coded as *DMBT1*^*SR47*^ genotype also showed no significant effect on age at diagnosis.

## Discussion

Previous work has shown the importance of *DMBT1* in the aetiology of CD using studies of knockout mice and genetic association of the *DMBT1*^*SR47*−^ deletion allele within the gene and CD. However, the genetic association had not been tested on another case–control cohort and had a relatively small sample size, and such association studies are prone to false-positive results through differential bias or chance effects. Furthermore, the effect size observed (OR=1.75) is larger than most effect sizes identified by GWAS^26^ and, if correct, could potentially be of clinical importance.

We conducted this study to try and replicate a previous genetic association study of the *DMBT1*^*SR47*−^ deletion with CD. We used a combination of publicly available data, generated as part of the Wellcome Trust Case Control Consortium study of copy number variation, and data were generated on three case–control cohorts using paralogue ratio tests to type the *DMBT1*^*SR47*−^ deletion on a total of 2679 cases and 4088 controls. Comparisons between PRT raw data and arrayCGH data showed that while arrayCGH reflects copy number variation, correct normalisation is important to optimise the copy number calling, even when clear clusters of raw values are observed. After meta-analysis of our data, we did not replicate the original association,^13^ and this could be due to a number of reasons. It is possible that, because we focused on Northern European populations and the original study was conducted on an Italian sample, the *DMBT1*^*SR47*−^ deletion allele confers susceptibility to CD only in Italian populations, perhaps due to an interaction with diet. It is also possible that different diagnosis criteria were used, perhaps enriching for a particularly severe clinical phenotype in the original study, although there is no indication of this in the original study. However, the most likely explanation is that this was a false-positive result. It should be noted that in the original study, the genotype frequencies for the cases show an extreme deviation from Hardy-Weinberg Equilibrium, with an excess of heterozygotes (*P*=5 × 10^−4^, *χ*^2^ test with 1 d.f.), which we do not observe in our data. We conducted a test for heterogeneity across our data sets and including the original data previously published, which suggested that the original data set was from a distinct population (*P*=0.039) and combining all the data in a meta-analysis would be inappropriate.

Previous analysis of CNVs within the *DMBT1* gene has shown that the *DMBT1*^*SR47*−^ deletion is in fact part of a multiallelic CNV called CNV1, and that another CNV, called CNV2, is 3′ to CNV1 and also affects the number of SRCR domains.^20^ By using our PRT assays, we typed multiallelic copy number for both CNV1 and CNV2 on the Scottish, English and Danish case–control cohorts, and found no evidence of association. In this study, we use our copy number typing approaches to call the full spectrum of copy number variation at both CNV1 and CNV2. Given that the full range of copy numbers can be typed, we might expect more power to detect any association that was linearly dependent on copy number, but we do not detect such an effect for CNV1 nor CNV2, nor could we show any association with CNV1 copy number or CNV2 copy number.

One important feature of the *DMBT1*^*SR47*−^ deletion allele is that, as part of CNV1, it has a remarkably high mutation rate of 0.7–2.7% per generation.^20^ This has the consequence that *DMBT1*^*SR47*−^ deletions will be generated by recurrent mutation, thereby eroding linkage disequilibrium with neighbouring SNP alleles. A recent study has identified a SNP allele associated with CD within *DMBT1* at genome-wide significance levels.^27^ It is unclear why this allele has not been identified by GWAS studies, and indeed it may not be in LD with SNPs assayed by GWAS studies; so further research is needed to dissect the nature of this association. Our results in this study do not exclude an association of single nucleotide variation within *DMBT1* and CD, nor do they exclude a role for *DMBT1* in CD, which has previously been suggested by the Dmbt1 knockout mouse. Indeed, *DMBT1* shows increased expression in the intestinal mucosa in CD patients, and this increased expression is dependent on NOD2 activation, because this response is abolished in CD patients homozygous for a *NOD2* SNP allele causing a *NOD2* frameshift, an allele also associated with CD.^28^ Given the role of DMBT1 in binding bacteria,^29, 30^ it seems reasonable to assume that the *DMBT1*^*SR47*−^ deletion allele encodes a protein that has an altered interaction with the intestinal flora, and mediates its effect via its interactions with bacteria. However, our study has excluded a role for the extensive copy number variation of *DMBT1* in strongly modifying the susceptibility to CD.

## Figures and Tables

**Table 1:** Association analysis of *DMBT1* ^*SR47*^ genotype with Crohn's disease

*Population*	*Cohort*	DMBT1^SR47−/−^ *number (frequency)*	DMBT1^SR47+/−^ *number (frequency)*	DMBT1^SR47+/+^ *number (frequency)*	Total	*Fisher's exact test* P-*value*	*Odds ratio*
Scottish	CD	5 (0.01)	57 (0.16)	286 (0.82)	348	0.93	0.97 (0.68–1.39)
	Controls	3 (0.01)	61 (0.18)	276 (0.81)	340		
English	CD	7 (0.01)	178 (0.19)	761 (0.80)	946	0.20	1.19 (0.91–1.56)
	Controls	2 (0.00)	79 (0.16)	399 (0.83)	480		
Danish	CD	5 (0.03)	34 (0.22)	116 (0.75)	155	0.09	1.53 (0.95–2.46)
	Controls	4 (0.02)	26 (0.15)	144 (0.83)	174		
WTCCC	CD	16 (0.01)	226 (0.18)	988 (0.80)	1230	0.76	1.05 (0.90–1.23)
	Controls	41 (0.01)	535 (0.17)	2517 (0.81)	3093		
Total	CD	33 (0.01)	495 (0.19)	2151 (0.80)	2679	0.22	1.07 (0.96–1.21)
	Controls	50 (0.01)	701 (0.17)	3337 (0.82)	4087		

**Table 2:** Association analysis of *DMBT1* CNV1 copy number with Crohn's disease

	*Scottish*		*English*		*Danish*	
*CNV1 copy number*	*CD*	*Controls*	*CD*	*Controls*	*CD*	*Controls*
0	5	3	7	2	5	4
1	57	61	178	79	34	26
2	275	263	731	387	114	139
3	11	12	30	11	2	5
4	0	1	0	1	0	0
*n*	348	340	946	480	155	174
Failed	2	0	3	0	1	5
Mean	1.84	1.84	1.83	1.85	1.73	1.83
SD	0.48	0.49	0.47	0.44	0.54	0.49
OR (95% CI) per copy	0.979 (0.717–1.335)		0.886 (0.694–1.126)		0.673 (0.435–1.028)	
*P* (log reg)	0.891		0.323		0.070	

**Table 3:** Association analysis of *DMBT1* CNV2 copy number with Crohn's disease

	*Scottish*		*English*		*Danish*	
*CNV2 copy number*	*CD*	*Controls*	*CD*	*Controls*	*CD*	*Controls*
1	0	0	1	0	0	0
2	13	8	14	11	2	9
3	29	34	98	48	12	21
4	82	79	229	110	30	44
5	78	83	319	125	46	43
6	70	69	164	104	36	33
7	37	26	67	51	16	11
8	23	18	35	23	10	11
9	11	10	3	4	4	5
10	2	1	2	4	0	0
11	1	0	0	0	0	0
*n*	346	328	932	480	156	177
Failed	4	12	17	0	0	2
Mean	5.27	5.15	4.95	5.15	5.34	4.97
SD	1.68	1.56	1.31	1.49	1.46	1.63
OR	1.05 (0.95–1.15)		0.901 (0.832–0.976)		1.169 (1.017–1.348)	
*P*	0.329		0.0103		0.0297	

**Table 4:** Multiple linear regression analysis testing association of CNV with age of onset

*Variable*	*B*	*B (SE)*	t*-Statistic*	P
Intercept	30.4	7.31	4.15	4.3 × 10^−5^
Sex (reference=female)	−4.76	1.83	−2.61	0.0097
CNV1	0.804	1.62	0.43	0.67
CNV2	0.0113	0.513	0.022	0.98

*N*=306, 7 omitted due to missing data.

Predictor variables were sex, CNV1 copy number and CNV2 copy number, with age at diagnosis the dependent variable. The values for the effect size (B) with its SE are given, together with the corresponding *t*-statistic used to test whether the value of B is significantly different from zero. The *P*-value of that test is given in the rightmost column.
