# Supplementary material for: Copy number variation of scavenger-receptor cysteine-rich domains within DMBT1 and Crohn's disease
Source: Eur J Hum Genet. 2016 Jan 27;24(9):1294–300. doi: 10.1038/ejhg.2015.280 (PMC4851238; doi:10.1038/ejhg.2015.280)
Supplement: Supplementary Tables [file ejhg2015280x1.docx]

**Supplementary table 1 Control calibrator DNA samples**

| HapMap sample | CNV1 copy number | CNV2 copy number |
| --- | --- | --- |
| NA18956 | 0 | 2 |
| NA18555 | 1 | 2 |
| NA12752 | 1 | 5 |
| NA18517 | 2 | 2 |
| NA10855 | 2 | 4 |
| NA12044 | 2 | 5 |
| NA07056 | 2 | 6 |
| NA18507 | 3 | 3 |

**Supplementary table 2 – Analysis of CNV1 case-control association status using CNVtools**

| **Comparison** | **Clustering Q value** | **-2 log likelihood** | **P value** |
| --- | --- | --- | --- |
| English CD vs controls | 10.397 | 0.612401 | 0.43 |
| Scottish CD vs controls | 9.311 | 0.005045 | 0.94 |
| Danish CD vs controls | 9.013 | 2.704464 | 0.10 |

**Supplementary table 3 – CNV2 comparisons**

| **Comparison** | **Clustering Q value** | **-2 log likelihood** | **P value** |
| --- | --- | --- | --- |
| English CD vs controls | 3.804 | 4.806162 | 0.028 |
| Scottish CD vs controls | 3.813 | 0.378274 | 0.53 |
| Danish CD vs controls | 3.979 | 10.68545 | 0.0011 |
