# Supplementary figures and images for: Copy number variation of scavenger-receptor cysteine-rich domains within DMBT1 and Crohn's disease
Source: Eur J Hum Genet. 2016 Jan 27;24(9):1294–300. doi: 10.1038/ejhg.2015.280 (PMC4851238; doi:10.1038/ejhg.2015.280)

English CNV1

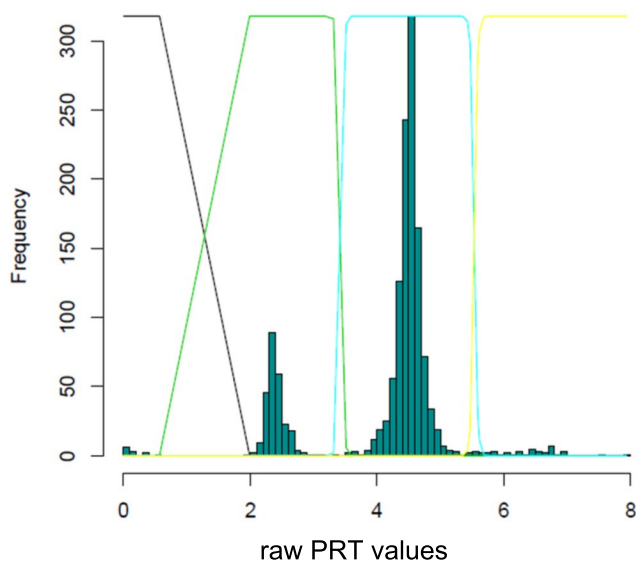

English CNV2

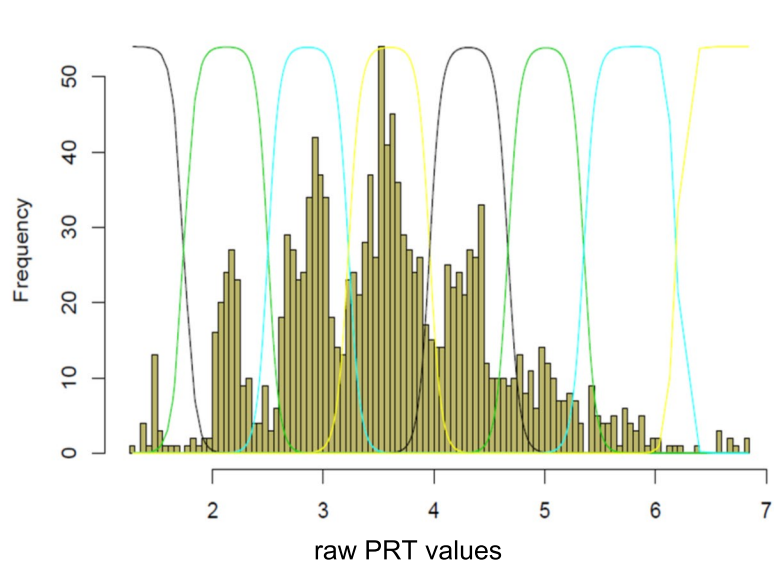

Scottish CNV1

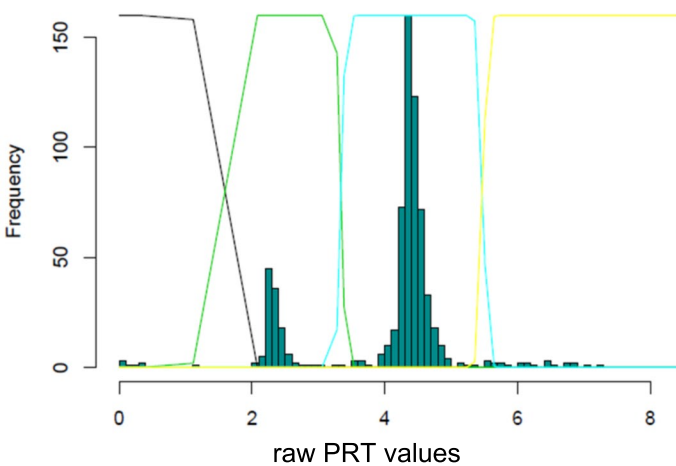

Scottish CNV2

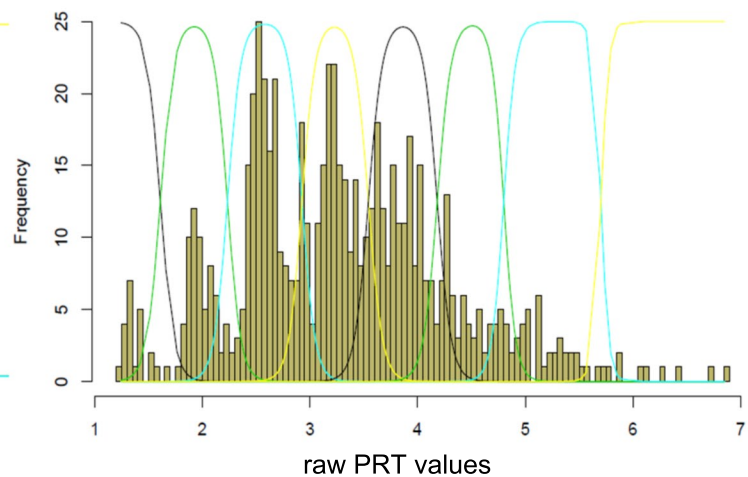

Danish CNV1

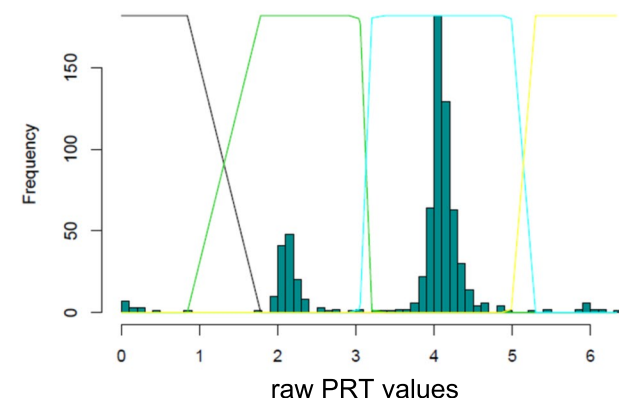

Danish CNV2

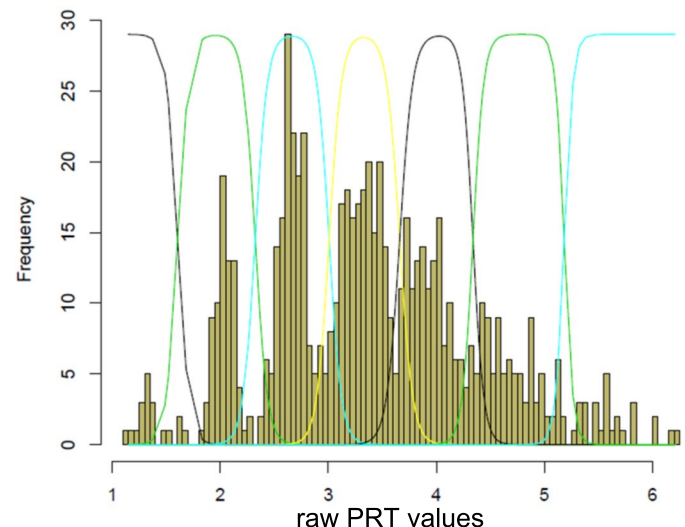

Supplement: Supplementary Figure 1 [file ejhg2015280x2.pdf]

Crohn's disease cases

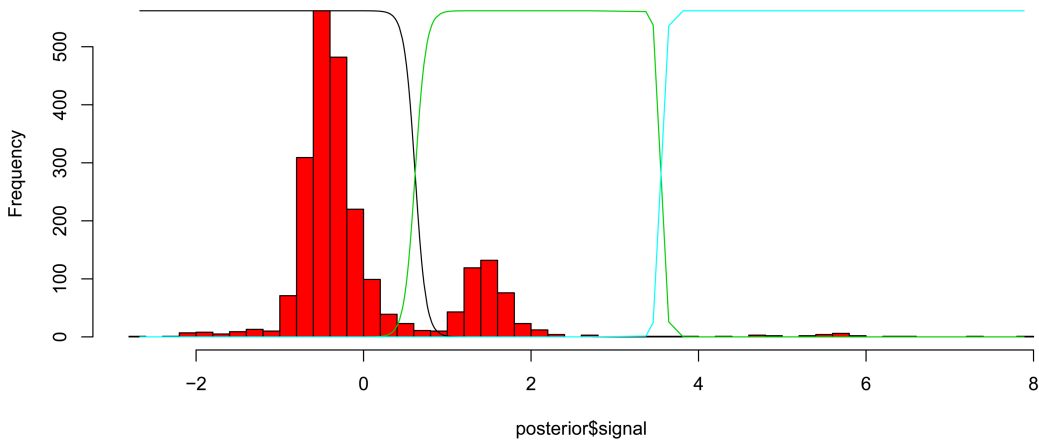

1958 cohort controls

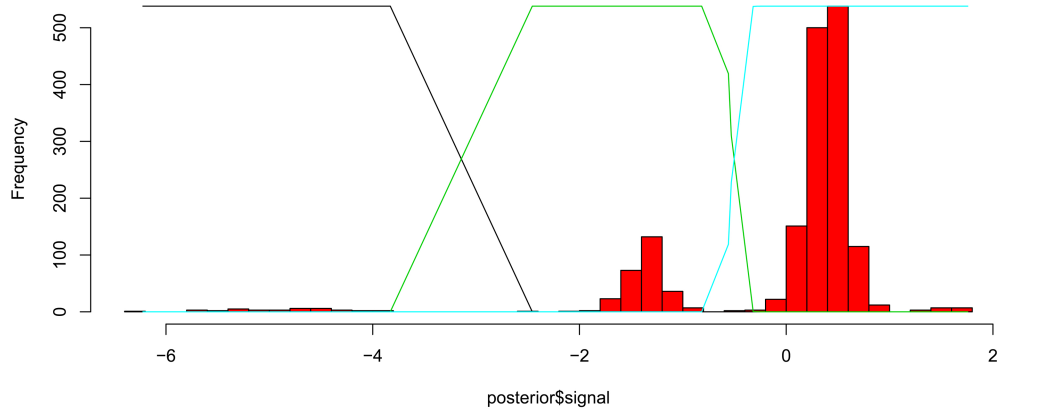

NBS controls

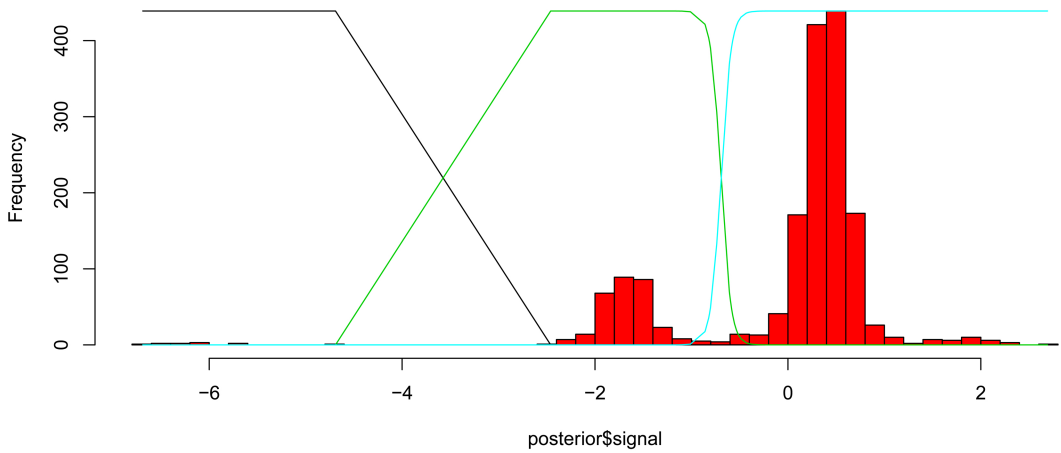

Supplement: Supplementary Figure 2 [file ejhg2015280x3.pdf]
