# Supplementary material for: Copy number variation of scavenger-receptor cysteine-rich domains within DMBT1 and Crohn's disease
Source: Eur J Hum Genet. 2016 Jan 27;24(9):1294–300. doi: 10.1038/ejhg.2015.280 (PMC4851238; doi:10.1038/ejhg.2015.280)
Supplement: Supplementary Figure Legends [file ejhg2015280x4.docx]

**Supplementary figure 1 Quality of CNV clustering by PRT**

This shows, for each cohort and CNV, a histogram of PRT copy number values (x-axis), normalised so that the entire distribution has a standard deviation of 1, to optimise Gaussian mixture model fitting. The lines show the fitted Gaussian mixture model separating each peak in the histogram.

**Supplementary figure 2 Quality of CNV1 clustering on the WTCCC cohort**

This shows, for three WTCCC cohorts, a histogram of the first principal component of 12 aCGH probes, normalised using the normalised1 method. The lines show the fitted Gaussian mixture model separating each peak in the histogram. Note that the direction of signal of the first principal component is arbitrary, so for the Crohn’s disease cases the histogram is reversed, as compared to the control sample histograms.
